# Intracameral Microimaging of Maturation of Human iPSC Derivatives into Islet Endocrine Cells

**DOI:** 10.1177/09636897211066508

**Published:** 2022-02-14

**Authors:** Kaixuan Zhao, Yue Shi, Jia Yu, Lina Yu, Amber Mael, Yuxin Li, Anthony Kolton, Thomas Joyce, Jon Odorico, Per-Olof Berggren, Shao-Nian Yang

**Affiliations:** 1 The Rolf Luft Research Center for Diabetes and Endocrinology, Karolinska Institutet, Karolinska University Hospital L1, Stockholm, Sweden; 2 Regenerative Medical Solutions, Inc., Madison, WI, USA; 3 National Engineering Laboratory for Druggable Gene and Protein Screening, Northeast Normal University, Changchun, China

**Keywords:** the anterior chamber of the eye (ACE), backscattering, human induced pluripotent stem cells (hiPSCs), in vivo confocal microscopy, surrogate islet, transplantation, vascularization

## Abstract

We exploited the anterior chamber of the eye (ACE) of immunodeficient mice as an ectopic site for both transplantation and microimaging of engineered surrogate islets from human induced pluripotent stem cells (hiPSC-SIs). These islets contained a majority of insulin-expressing cells, positive or negative for PDX1 and NKX6.1, and a minority of glucagon- or somatostatin-positive cells. Single, non-aggregated hiPSC-SIs were satisfactorily engrafted onto the iris. They underwent gradual vascularization and progressively increased their light scattering signals, reflecting the abundance of zinc-insulin crystal packaged inside mature insulin secretory granules. Intracameral hiPSC-SIs retrieved from recipients showed enhanced insulin immunofluorescence in correlation with the parallel increase in overall vascularization and light backscattering during the post-transplantation period. This approach enables longitudinal, nondestructive and intravital microimaging of cell fates, engraftment, vascularization and mature insulin secretory granules of single hiPSC-SI grafts, and may offer a feasible and reliable means to screen compounds for promoting in vivo hiPSC-SI maturation.

## Introduction

The establishment of human induced pluripotent stem cell (hiPSC) technology has evoked significant efforts in manufacturing surrogate islets (SIs) from hiPSCs in vitro for diabetes therapy^1–9^. However, hiPSC-SIs have yet to be used to treat patients with diabetes as they show partial function in vitro due to the presence of immature or undesirable cell populations, and exhibit delayed maturation after transplantation and in some cases may demonstrate unreliable safety profiles in vivo^10–16^. Importantly, it has not been feasible to analyze and understand these limitations and what happens as hiPSC-SI grafts evolve after transplantation in a noninvasive, serial intravital manner with a high spatiotemporal resolution owing to typical approaches by which large numbers of SIs are densely packed in optically inaccessible sites^4,6,11,17–22^. Here, we show that intravital microscopy enables serial, noninvasive microimaging of cell fates, engraftment, vascularization and insulin secretory capacity of single hiPSC-SIs when transplanted into the anterior chamber of the eye (ACE) of immunodeficient mice. This new method establishes a basis for understanding molecular and cellular events in vivo and for developing effective mechanism-based means to manipulate hiPSC-SIs for therapeutic needs in the clinic.

## Materials and Methods

### Generation of hiPSC-SIs

Undifferentiated hiPSC line NCRM-1 (Lonza/NIH, Walkersville, MD, USA) was cultured using mTeSR1 (Stem Cell Technologies, Vancouver, BC, Canada) on Growth Factor Reduced Matrigel (Corning, Corning, NY, USA) at 37°C in a humidified 5% CO_2_ incubator. Cells were passaged every 3-4 days by single-cell dispersion using TrypLE (Invitrogen, Carlsbad, CA, USA).

To initiate differentiation, undifferentiated cells were single-cell dispersed and seeded at 5 x 10^5^ cells/ml into Matrigel coated Transwell (Corning) culture plates. Cells were cultured for 24 hours in mTeSR1 and then cultured under differentiation medium conditions for 7 stages. These medium conditions were set by supplementing DMEM/F-12 with 100 ng/ml Activin A, 100 ng/ml bFGF, 10 ng/ml BMP4 and 3 μM CHIR99021 (Tocris, Ellisville, MO, USA), 2% BSA (MilliporeSigma, Burlington, MA, USA), 1x NEAA (Invitrogen), 1x Glutamax (Invitrogen) at Stage 1 (3 days); 1x ITS-X (Invitrogen), 50 ng/ml FGF7, 10 mM Nicotinamide (MilliporeSigma) at Stage 2 (3 days); 1x ITS-X, 2 μM Retinoic Acid (MilliporeSigma), 300 ng/ml Noggin at Stage 3 (2 days); 1x ITS-X, 50 ng/ml EGF, 50 ng/ml FGF7, 10 mM Nicotinamide at Stage 4 (4 days); 1x ITS-X, 10 ng/ml FGF7, 300 ng/ml Noggin, 10 mM Nicotinamide Stage 5 (4 days); 1x B27 without Insulin (Invitrogen), 10 mM Nicotinamide, 42.8 ng/ml Exendin-4 (MilliporeSigma), 10 μM Alk5i, 10 ng/ml FGF7, 20 ng/ml EGF, 100 nM Gamma Secretase Inhibitor (MilliporeSigma) at Stage 6 (6 days); and 1x B27 without Insulin, 10 μM Alk5i, 25 μM Forskolin, 10 μM ZnSO_4_ (Fluka, Milwaukee, WI, USA), 1 μM T3 (MilliporeSigma), 10 ng/ml Heparin (MilliporeSigma), 10 mM L-Glutathione (MilliporeSigma), 10 μM LY294002 (Invitrogen) at Stage 7 (8 days). At the beginning of stage 7, cells are removed from the Transwell culture plates, resized and placed into Ultra Low Attachment flasks (Corning) for further maturation in suspension. The resultant hiPSC-SIs were subjected to in vitro quality testing and used for intracameral transplantation when they reached the end of the final stage of differentiation, that is, Stage 7. All reagents were obtained from R&D Systems unless otherwise stated.

### Flow Cytometry

Differentiated cells at specific stages were fixed with 2% methanol-free formaldehyde in PBS for 30 minutes. Cells were permeabilized with 0.1% BSA and 0.1% saponin in PBS for 1 hour and stained with conjugated antibodies against insulin (Cell Signaling, Danvers, MA, USA), PDX1, NKX6.1 and glucagon (BD, San Jose, CA, USA) for 1 hour and subjected to Flow cytometry.

### Insulin Secretion Assay

Approximately 50 hiPSC-SIs or human cadaveric islets (Prodo Labs, Aliso Viejo, CA, USA) were collected and placed in tissue culture inserts (MilliporeSigma) in a 24-well plate. All cells were first washed with KRB buffer (128 mM NaCl, 5 mM KCl, 2.7 mM CaCl_2_, 1.2 mM MgSO_4_, 1 mM Na_2_HPO_4_, 1.2 mM KH_2_PO_4_, 5 mM NaHCO_3_, 10 mM HEPES (Gibco, Waltham, MA, USA) and 0.1% BSA. Cells were then incubated in KRB containing 2.5 mM glucose at 37ºC for one hour, after which this solution was discarded and replaced with fresh 2.5 mM glucose KRB solution. After an additional hour, the supernatant was collected. For measuring glucose-stimulated insulin secretion, 25 mM glucose KRB was added for the next hour. Thereafter, the supernatant was again collected. Cells were washed with fresh KRB during each solution change. Cells were then single-cell dispersed with TrypLE and counted with the TC-20 (BioRad, Hercules, CA, USA). Supernatants from the low and high glucose challenges were quantified with a human c-peptide ELISA (R&D Systems, Minneapolis, MN, USA), and cell counts were used to normalize insulin secretion.

### Animals

NOD-*scid* IL2Rgamma^null^ mice at 8-10 weeks of age were purchased from Charles River Laboratories (Sulzfeld, Germany). They were maintained on a regular light-dark cycle (lights on at 07.00 h and off at 19.00 h) in temperature and humidity-controlled rooms, had free access to food pellets and tap water, and randomly selected as hiPSC-SI transplant recipients.

### hiPSC Transplantation

Recipient mice were individually subjected to induction of general anesthesia in a veterinary anesthesia induction chamber containing isoflurane followed by inhalation of a mixture of 2.5% isoflurane and 40% oxygen via a nose mask. The head and eyeball of anesthetized mice were immobilized with a head holder and stabilized with an eyeball holder, respectively, for transplantation surgery^23^. Next, hiPSC-SIs are gently aspirated into a glass micropipette, referred to as donor tissue-delivering microcannula, connected by tygon tubing to a threaded plunger syringe. The tip of the donor tissue-delivering microcannula is beveled and lightly heat-polished. The microcannula tip opening has an interior diameter ranging from 100 to 300 μm. Subsequently, a tiny corneal hole is made by carefully inserting the tip of an insulin syringe needle (29G) through the cornea. Then immediately, the tip of the microcannula preloaded with hiPSC-SIs was cautiously inserted into the corneal hole and preloaded hiPSC-SIs were gently injected into the ACE. Finally, the tip of the glass micropipette is prudently and slowly extracted out of the corneal hole to avoid escape of hiPSC-SIs from the hyperbaric ACE. After transplantation surgery, the recipients were released from head and eyeball holders and kept lying on its side before recovery from anesthesia.

### In Vivo Stereomicroscopy

The eyeball of anesthetized and immobilized recipient mice was positioned under an upright stereomicroscope (Leica Microsystems Heidelberg GmbH, Mannheim, Germany) and tilted to an orientation suitable for stereomicroscopy by fine adjustment of head and eyeball holders. Intravital stereomicroscopy and photography were performed on the same intracameral hiPSC-SIs at different time points post-transplantation.

### In Vivo Confocal Microscopy

Anesthetized and immobilized recipient mice were placed under an upright Leica DM6000 CFS microscope equipped with a Leica TCS SP5 II confocal laser-scanner (Leica Microsystems). The eyeball was tilted to a proper orientation for microscopic imaging as described above and the cornea was covered with Viscotears (Théa Laboratories, Clemont-Ferrand, France). Subsequently, a long-distance water-dipping lenses (Leica HXC APO L10×/0.3 W U-V-I) was immersed into Viscotears on the cornea and focused on a hiPSC-SI of interest. The backscattering signal was registered when 633 nm laser light was used to illuminate the ACE and the detection wavelength of reflected light was set at 630-640 nm^23^. For visualization of vasculatures in hiPSC-SI grafts and their attached iris, recipient mice received injection of 0.4 μmol/L Qtracker 565 (Life Technologies, Carlsbad, CA, USA) in 100 µL saline into the tail vein. Intravascular Qtracker 565 was excited by a 488 nm laser line and the resultant emissions were collected at 550-585 nm. Z-stack images were obtained every 2-3 µm and analyzed with Leica Confocal Software (Leica Micosystems) and Volocity (PerkinElmer, Waltham, MA, USA). The relative intensity of intracameral hiPSC-SI backscatter was obtained by normalizing the mean voxel intensity of intracameral hiPSC-SI backscatter to that of iris backscatter. The vascular density of intracameral hiPSC-SIs was defined as the percentage of vascular volume in intracameral hiPSC-SI volume estimated from stack imaging of intracameral hiPSC-SI backscatter. One hiPSC-SI per recipient mouse was microimaged to acquire clear images before the significant leaking of intravenously injected Qtracker 565 and to follow ethical guidelines. Mice were kept lying on a thermostat bed to maintain its body temperature at 37°C during the course of an imaging experiment.

### Immunocytochemistry and In Vitro Confocal Microscopy

Recipient mice were anesthetized with a Hypnorm/sterile water/Midazolam Hameln mix (1:2:1) (100 μl/10 g bodyweight, i.p.) and perfused transcardially with 0.9% saline supplemented with heparin (1 IU/ml) followed immediately by 4% paraformaldehyde. Then, eyeballs carrying hiPSC-SI transplants were rapidly but carefully removed and postfixed in 4% paraformaldehyde for 90-120 min. In addition, a fraction of pre-transplanted hiPSC-SIs of every batch were fixed in 4% paraformaldehyde for 90-120 min. Both postfixed eyeballs and fixed hiPSC-SIs were cryoprotected by sequential immersion in 20% sucrose for 2 h and 30% sucrose overnight. Thereafter, cryoprotected specimens were embedded in optimal cutting temperature (OCT) compound and frozen. They were either sectioned with a Cryostat into 20 µm sections or stored at -80°C for later use^24^.

For immunofluorescence labelling, specimens were permeabilized with 0.1% Triton-X 100 at room temperature for 10 min and then incubated with blocking solution at room temperature for 60 min. Subsequently, some specimens were triple-labeled with rabbit polyclonal antibody to glucagon (1:500; BioGenex, Fremont, CA, USA), guinea pig polyclonal antibody to insulin (1:1000, Dako North America, Carpinteria, CA, USA) and rat monoclonal antibody to somatostatin (1:1000; Novus Biologicals, Centennial, CO, USA). The antibody incubation was performed at 4°C overnight. The sections were then subjected to 60 min incubation with goat anti-rabbit, goat anti-guinea pig, and goat anti-rat IgG coupled to Alexa 488, Alexa 633, and Alexa 546 (1:1000; Molecular Probes, Eugene, OR, USA) at room temperature. Finally, specimens were mounted in Prolong Gold Mountant with DAPI (Molecular Probes) under coverslips and visualized with a Leica TCS SP8 X confocal laser-scanner equipped with a white light and 405 nm pulsed laser and connected to a Leica DMi 8 microscope (Leica Microsystems). Optical sections were captured using Leica HC PL APO 63x/1.30 GLYC CORR CS2 objective. Alexa 488, Alexa 633, and Alexa 546 linked to goat anti-rabbit, anti-guinea pig, and anti-rat IgG as well as DAPI were excited by a 488, 633, 546, and 405 nm laser line, respectively, and the resultant emissions were collected at 500-540, 660-730, 560-630, and 410-480 nm, respectively. Every set of immunocytochemistry experiments was performed with pretransplanted hiPSC-SIs as control and hiPSC-SIs retrieved from the ACE, both of which came from the same batch of hiPSC-SIs. The same parameters and settings were used for imaging the labeled cells from batch to batch. The confocal images were analyzed with Leica Confocal Software (Leica Microsystems) and Volocity (PerkinElmer).

### Statistical Analysis

All data are expressed as mean ± SEM. One-way ANOVA followed by least significant difference (LSD) test, the non-parametric Mann-Whitney *U* test and Kruskal-Wallis test were used to determine statistical significance. *P*-values below 0.05 were considered statistically significant.

## Results

### In Vitro Quality Evaluation of Pretransplanted hiPSC-SIs

**Figure 1.**
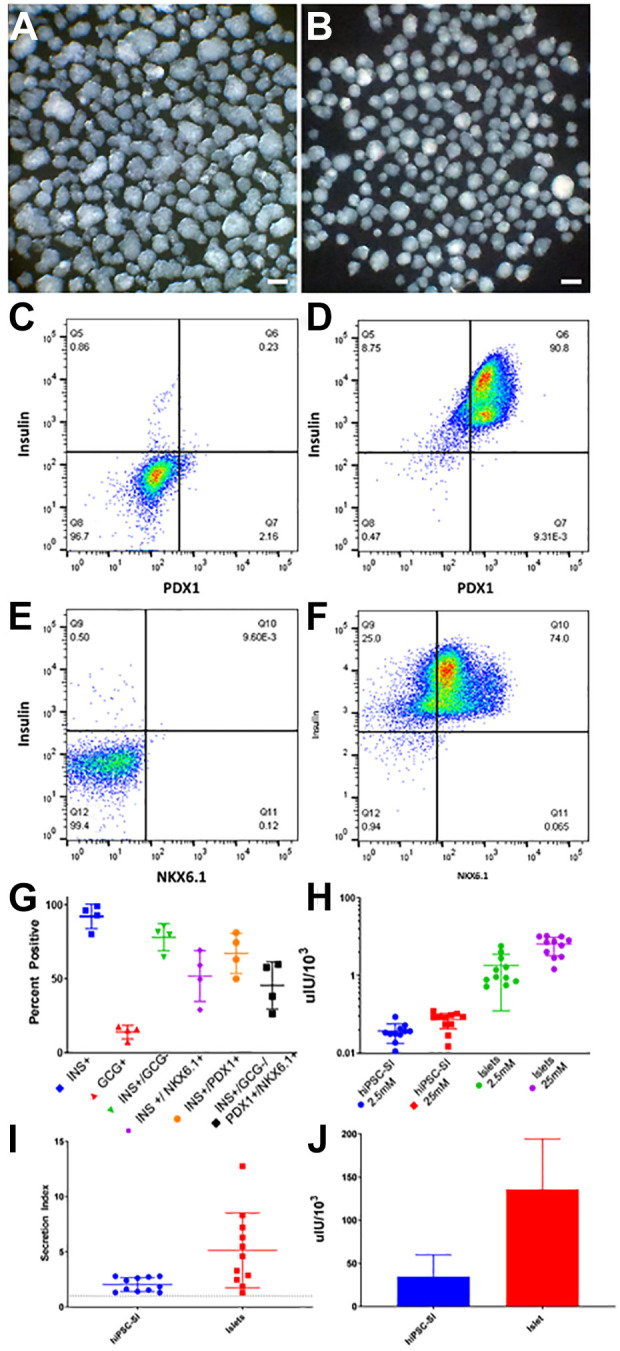
In vitro quality testing for pretransplanted hiPSC-SIs. (A and B) Stereomicroscopic photographs showing original (A) and selected in vitro-generated hiPSC-SIs for intracameral transplantation (B). (C-F) Representative flow cytometry plots illustrating insulin costaining with PDX1 and NKX6.1 (C), unstained cell population gated for insulin and PDX1 (D), insulin+/PDX1+ cell population (E) and unstained cell population gated for Insulin and NKX6.1 (F) in hiPSC-SIs. (G) Summary data from flow cytometric analysis showing percentages of insulin-positive (INS+), glucagon-positive (GCG+), insulin-positive/glucagon-negative (INS+/GCG-), insulin-positive/NKX6-1-positive (INS+/NKX6.1+), insulin-positive/PDX1-positive (INS+/PDX1+), insulin-positive/glucagon-negative/PDX1-positive/NKX6.1-positive (INS+/GCG-/PDX1+/NKX6.1+) cell populations in hiPSC-SIs. (H) Glucose stimulated insulin secretion of hiPSC-SIs compared to cadaver derived islets, presented as uIU secreted per 1000 cells. (I) Secretion Index (insulin release in 25 mM glucose / insulin release in 2.5 mM glucose) for hiPSC-SI and cadaver derived islet in H. (J) Total insulin content of hiPSC-SIs as measured by C-peptide ELISA, presented as uIU secreted per 1000 cells. The in vitro quality testing was done for all the batches of hiPSC-SIs used in the present study.

Clinically available β cell replacement therapies are currently limited due to the shortage of donor islets, their inconsistent availability and quality, as well as the inability to noninvasively, serially monitor the graft at high spatiotemporal resolution^25^. Therefore, we sought to establish a method for nondestructive, intravital and serial monitoring via the ACE using hiPSC-SIs, which may serve as an abundant, reproducible source of human β cells. To that end, we first generated hiPSC-SIs in vitro using the established hiPSC line NCRM-1 and then conducted a series of in vitro quality tests (Fig. 1A-J). Stereomicroscopy showed that hiPSC-SIs were spherical, elongated or irregular in shape, resembling native human islets (Fig. 1A, B). Their longest dimension ranged from 80 to 350 µm (Fig. 1A). The hiPSC-SIs of suitable size (< 250 µm in diameter) were selected for intracameral transplantation (Fig. 1B). Flow cytometry revealed that hiPSC-SIs consisted of 93% insulin-positive, 78% insulin-positive/glucagon-negative and 41% insulin-positive/glucagon-negative/PDX1-positive/NKX6.1-positive cells (Fig. 1C–G). Insulin secretion assay showed that hiPSC-SIs displayed a 2.28-fold increase in insulin secretion following incubation with 25 mM glucose (Fig. 1H, I) and that hiPSC-SIs retained 25% of insulin content of human cadaver islets (Fig. 1J).

### In Vivo and In Vitro Microimaging of Single hiPSC-SIs Implanted into the ACE

**Figure 2.**
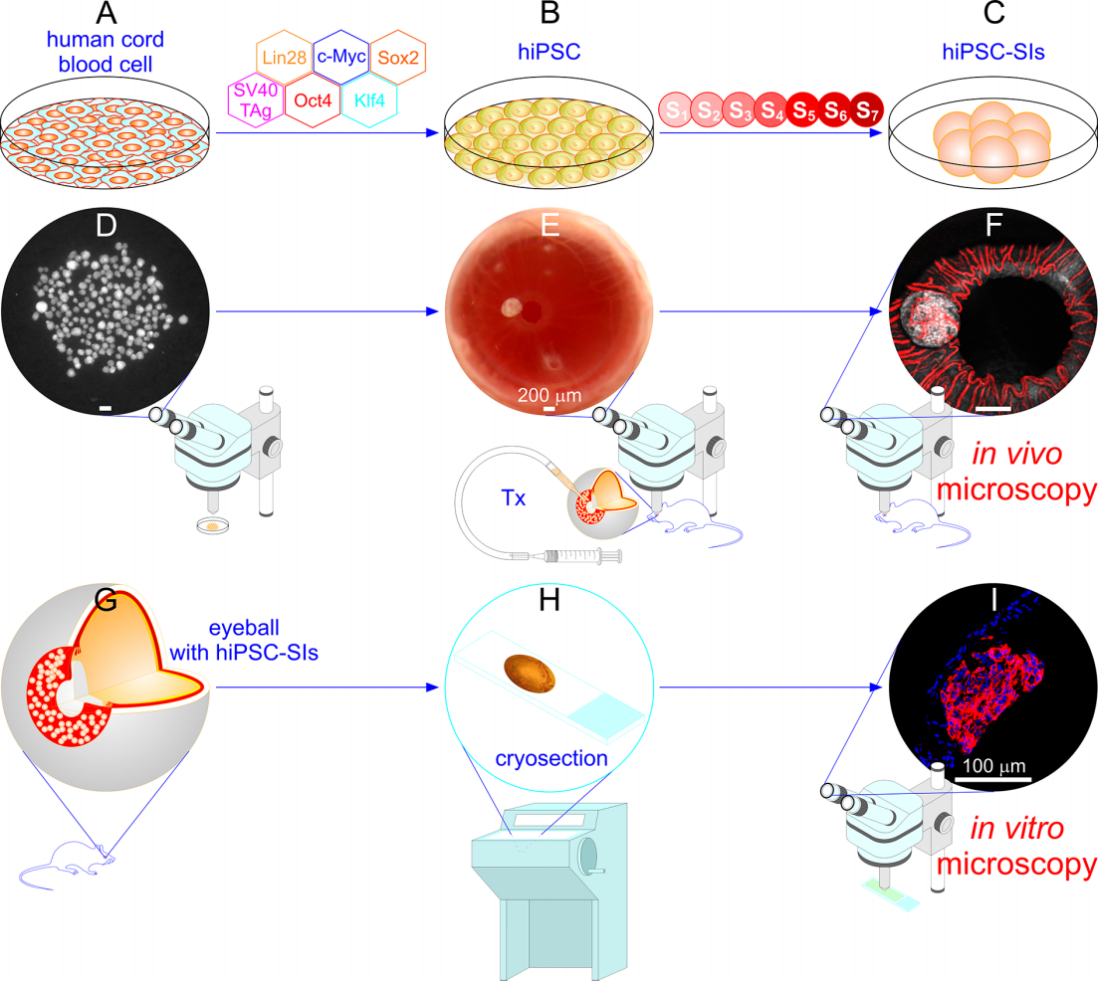
Schematic of in vivo microscopy of hiPSC-SIs transplanted into the mouse ACE complemented by in vitro microscopy of hiPSC-SIs retrieved from the mouse ACE. (A-F) In vivo microscopy consists of the three steps. First, hiPSC-SIs were generated by differentiating the undifferentiated hiPSC line NCRM-1, which was manufactured from human cord blood cells by ectopic expression of Oct4, Sox2, Klf4, c-Myc, Lin28 and SV40 TAg^48^ through seven differentiation stages (S1-S7) in cell culture devices (A-C). Second, hiPSC-SIs were transplanted into the ACE of NOD-*scid* IL2Rgamma^null^ mice under a stereomicroscope (Tx) (D and E). Third, intracameral hiPSC-SIs were intravitally and noninvasively microimaged under a stereomicroscope or a confocal microscope, respectively (F). (G–I) In vitro microscopy also includes the three steps. First, eyeballs containing hiPSC-SIs were removed from recipient mice under a stereomicroscope at month 3 post-transplantation (G). Second, eyeballs were fixed in 4% paraformaldehyde, frozen in OCT and cryosectioned at 20-µm thickness (H). Third, sections were immunofluorescently labelled with specific antibodies to islet hormones and subjected to in vitro confocal microscopy (I). Stereomicroscopic photographs showing hiPSC-SIs of suitable size (< 250 µm in diameter) prior to transplantation (D). A stereo microimage illustrating hiPSC-SIs implanted into the mouse ACE (E). A confocal laser scanning micrograph showing the backscatter image (grey) merged with the confocal fluorescence image (red) of an hiPSC-SI engrafted on the iris of a recipient mouse after bolus injection of Qtracker 565 into the lateral tail vein (F). A scheme illustrating an eyeball with hiPSC-SI grafts (G). A scheme depicting a cryosection of an eyeball containing a hiPSC-SI graft (H). A confocal laser scanning micrograph showing an overlay of insulin immunofluorescence (red) with DAPI fluorescence (blue) (I). hiPSC-SIs: human induced pluripotent stem cell-derived surrogate islets. S_1-7_: differentiation stage 1-7. Tx: transplantation.

To establish in vivo and in vitro microimaging of single hiPSC-SIs, 10-20 size-selected hiPSC-SIs were gently injected into the ACE through a glass micropipette connected to a syringe (Fig. 2D, E). Injected hiPSC-SIs rapidly adhered or attached to the iris. We deliberately aimed to scatter hiPSC-SIs in regions from the iris collarette to the pupil edge by controlling the position of the tip of hiPSC-SI delivery micropipette and injection pressure and speed to afford optimal visualization under a microscope and with naked eyes. In most cases, we observed single, non-aggregated hiPSC-SIs scattered in the desired regions of the iris of recipient mice. Within about 20 min following injection, hiPSC-SIs attached themselves to the iris by gravity. After a 2-day recovery, in vivo stereomicroscopy/confocal microscopy were performed to monitor the in vivo gross morphology, engraftment, vascularization and light scattering signals of hiPSC-SIs transplanted into the ACE (Fig. 2E, F). A representative image acquired by noninvasive in vivo stereomicroscopy shows a hiPSC-SI engrafted on the iris at 14 days post-transplantation (dpt) (Fig. 2E). A sample confocal micrograph illustrates typical vascularization and light scattering signals of an intracameral hiPSC-SI at 14 dpt (Fig. 2F). At the end point of experiments, recipient mice were sacrificed, and their eyeballs containing intracameral hiPSC-SI grafts were removed and cryosectioned (Fig. 2G, H). Cryosections were subjected to immunofluorescence labelling and in vitro confocal microscopy (Fig. 2H, I). An in vitro confocal micrograph shows sample insulin-immunofluorescence and DAPI fluorescence in a hiPSC-SI graft attached to the iris in an eyeball dissected out at 90 dpt (Fig. 2I). Overall, we established the present approach by combining transplantation (Tx) into the ACE and three imaging modalities: in vivo stereomicroscopy, in vivo confocal microscopy and in vitro confocal microscopy, hereafter abbreviated as ACE-Tx/in vivo SM, ACE-Tx/in vivo CM and ACE-Tx/in vitro CM, respectively. This approach enables transplantation of hiPSC-SIs to the desired regions of the iris where they were fixed and isolated, thereby allowing optimal noninvasive in vivo microscopy complemented by in vitro microscopy of individual intracameral hiPSC-SIs.

### Longitudinal Nondisruptive Microimaging of In Vivo Dynamic Changes in the Gross Morphology, Vascularization and Light Backscattering of Single hiPSC-SIs Engrafted in the ACE

**Figure 3.**
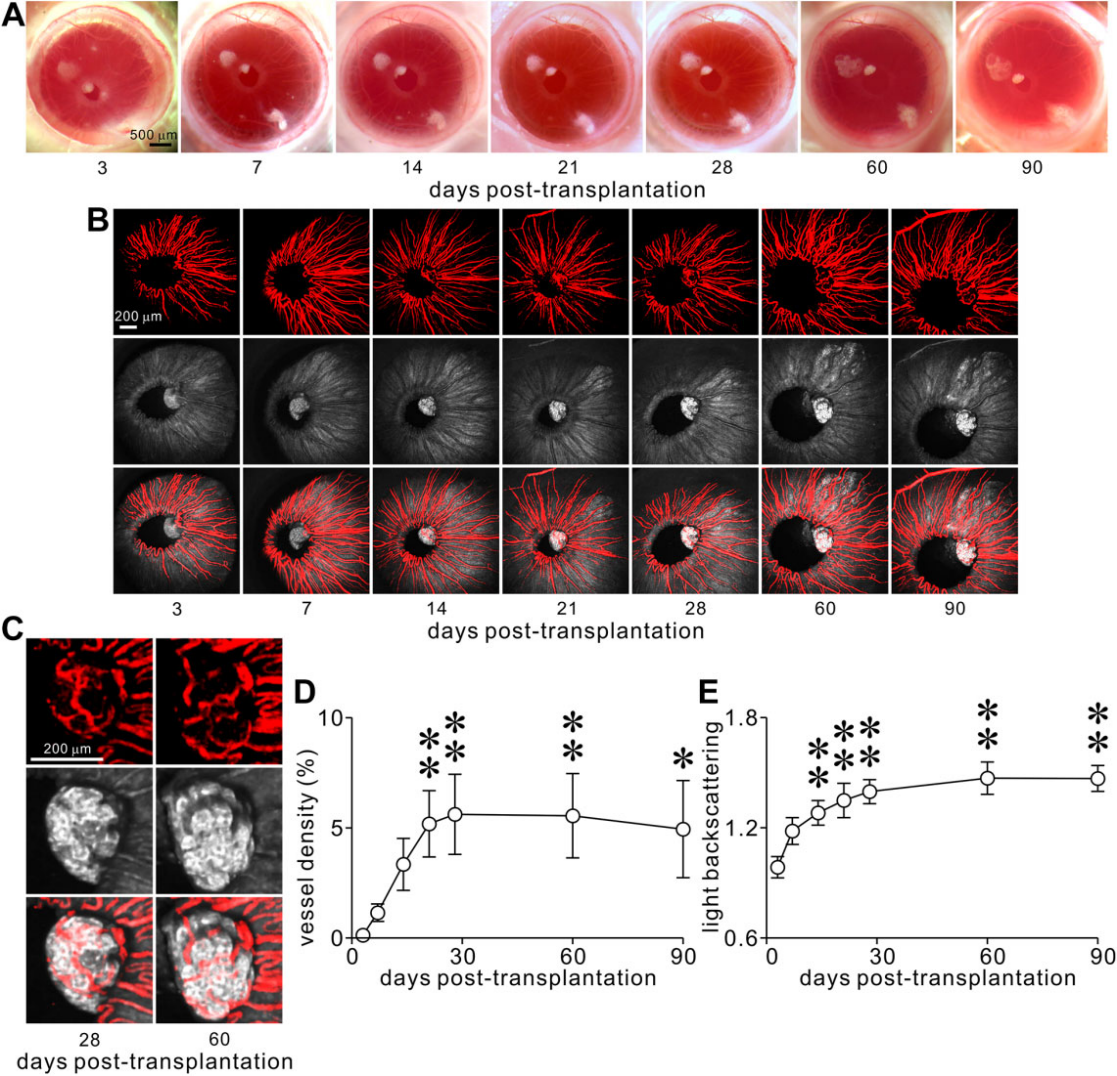
Longitudinal noninvasive microscopic characterization of the gross morphology of, the in vivo dynamics of vascularization of and the light backscattering from hiPSC-SIs transplanted into the mouse ACE. (A) Sample low magnification stereo micrographs of intracameral hiPSC-SIs scattered on the iris of recipient mice at day 3, 7, 14, 21, 28, 60 and 90 dpt. (B) Representative images of the Qtracker 565-labelled vasculatures (upper) in, backscattered light (middle) from an intact single intracameral hiPSC-SI and their overlays (lower) at 3, 7, 14, 28, 60 and 90 dpt. (C) High-magnification images of the Qtracker 565-labelled vasculatures (upper) in and backscattered light (middle) from an intact single intracameral hiPSC-SI and their overlay (lower) at 28 and 60 dpt. (D and E) Quantitative analysis of the vessel density (*n* = 8) (D) and light backscattering intensity (*n* = 8) (E) of intracameral hiPSC-SIs within 90-day post-transplantation period. Herein, *n* denotes the number of hiPSC-SIs, one of which comes from one recipient mouse. Each of four different batches of hiPSC-SIs was transplanted into two recipients. *P < 0.05 and **P < 0.01 *vs*. 3 dpt.

Upon general confirmation of the feasibility and merits of the approach, we directly used ACE-Tx/in vivo SM to nondestructively observe the gross morphology of hiPSC-SIs over time. ACE-Tx/in vivo SM showed that transplanted hiPSC-SIs rapidly adhered to the iris following injection into the ACE. Thereafter, the hiPSC-SIs gradually integrated with the iris and increased in size and changed their shape to varying degrees over the late post-transplantation period (Fig. 3A). Some transplanted hiPSC-SIs visibly altered their contour or became cystic within 90 dpt (Fig. 3A). In general, individual hiPSC-SIs displayed heterogeneity in their attachment, engraftment, survival and growth during the post-transplantation period. The results demonstrate that simple ACE-Tx/in vivo SM works well for noninvasively monitoring the in vivo dynamics of the gross morphology of intracameral hiPSC-SIs and helps identify single hiPSC-SIs for subsequent ACE-Tx/in vivo CM and ACE-Tx/in vitro CM.

Following ACE-Tx/in vivo SM, we noninvasively tracked the vascularization of single hiPSC-SIs with ACE-Tx/in vivo CM at 3, 7, 14, 21, 28, 60, and 90 dpt. In combination with in vivo vascular labeling with Qtracker 565, ACE-Tx/in vivo CM showed that the overall vascularization of single hiPSC-SIs gradually increased within the first 4 weeks post-transplantation and then remained relatively stable until the end of the observation period (Fig. 3B–D). Vascular density analysis showed that functional blood vessels sparsely emerged in single hiPSC-SIs by 3 dpt, especially in the regions of the islet which were directly connected to the iris (Fig. 3B–D). When imaged at 7 and 14 dpt, they became more and more enriched with perfusable vascularture (Fig. 3B–D). Thereafter, these vessels progressively spread forming appreciable microvascular networks throughout a single hiPSC-SI by 21 dpt (Fig. 3B–D). Subsequently, the vascular density increased during the first 4 weeks and plateaued at 28 dpt (Fig. 3B–D). The vascular density was significantly higher at 21, 28, 60, and 90 dpt than that at 3 dpt (*P* < 0.05). In addition to the increases in vascular density of intracameral hiPSC-SI grafts, the vascular networks also remodeled themselves by changing their distribution patterns (Fig. 3C). Thus, intracameral hiPSC-SIs can satisfactorily be vascularized within the first 4 weeks post-transplantation in the ACE. The data verify that ACE-Tx/in vivo CM can competently witness dynamic vascularization in single hiPSC-SIs scattered onto the iris surface at high resolution during the post-transplantation period.

In parallel with the intravital tracking of dynamic vascularization, we noninvasively monitored the in vivo dynamics of light scattering signals from single intracameral hiPSC-SIs by using ACE-Tx/in vivo CM. Average light scattering signals increased gradually within 28 dpt, peaked at 60 dpt and then remained unaltered until 90 dpt (Fig. 3B, C, E). Quantification shows that average light scattering signals progressively increased over the initial period from 3 to 28 dpt, plateaued at 60 dpt and then were sustained until the end of the experiment (Fig. 3B, C, E). The intensity of light scattering signals at 14, 21, 28, 60, and 90 dpt was significantly enhanced in comparison to that at 3 dpt (*P* < 0.01). The peak intensity of light scattering signals at 60 dpt was 1.49-fold higher than the basal one at 3 dpt (Fig. 3E). Furthermore, light scattering signals at all time points were not evenly distributed in different regions of an intracameral hiPSC-SI in terms of their intensity. Most regions displayed intense or moderate signals, some areas or small zones exhibited weak or no detectable signals within an individual hiPSC-SI (Fig. 3C). The results substantiate that ACE-Tx/in vivo CM is able to noninvasively and sensitively detect the in vivo dynamics of light scattering signals, which are an intrinsic optical indicator of zinc-insulin crystal packaged inside mature insulin secretory granules and reflect insulin secretory capacity^26–28^ of single hiPSC-SIs during the post-transplantation period.

### In Vitro Microscopic Comparison of Islet Hormone Immunofluorescence in Single hiPSC-SIs Before and After ACE Transplantation

**Figure 4.**
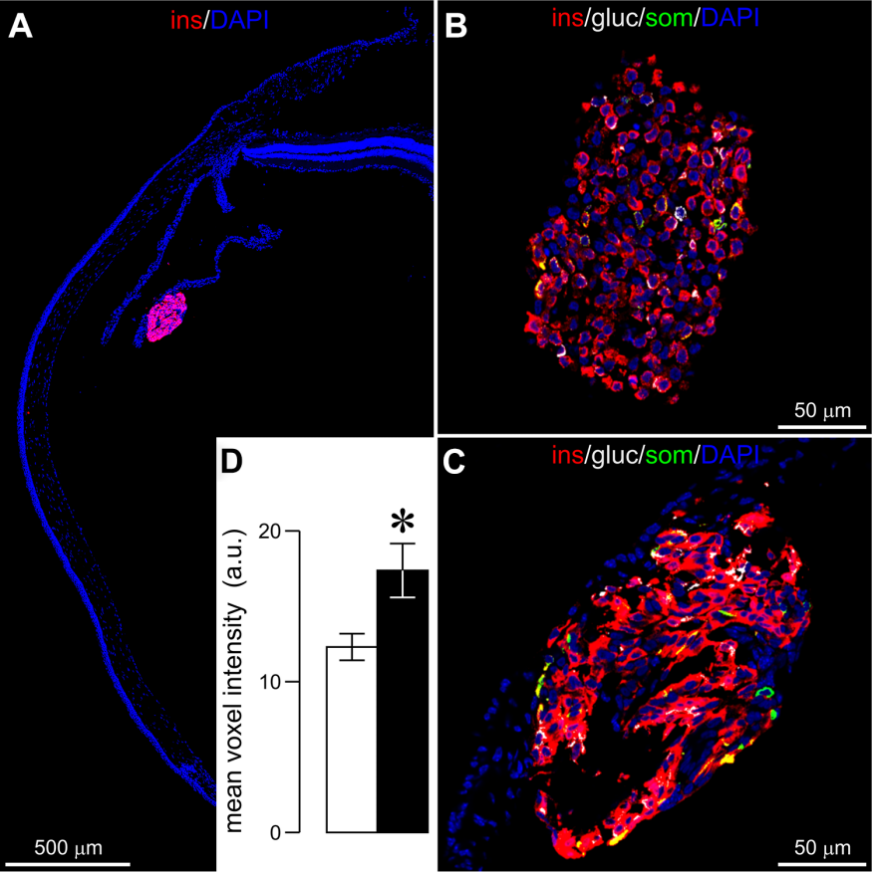
*In vitro* confocal microscopic characterization of islet hormone immunofluorescence 11 in single hiPSC-SIs before and after intracameral transplantation. A, Representative insulin 12 immunofluorescence (red) and DAPI fluorescence (blue) in the cryosection of an intact single 13 intracameral hiPSC-SI on the iris of an eyeball retrieved at 90 dpt. B, Sample insulin (red), 14 glucagon (white), somatostatin (green) immunofluorescence and DAPI fluorescence (blue) in a 15 cryosectioned hiPSC-SI before intracameral transplantation. C, An example image of insulin 16 (red), glucagon (white), somatostatin (green) immunofluorescence and DAPI fluorescence (blue) 17 in a cryosection of an intact single intracameral hiPSC-SIs retrieved at 90 dpt. D, Quantitative 18 analysis of insulin immunofluorescence in hiPSC-SIs not subjected to intracameral 19 transplantation as control (n = 8) and intraoclular hiPSC-SIs retrieved from eyeballs at 90 dpt (n 20 = 8. Herein, n denotes the number of hiPSC-SIs, one of which comes from one recipient mouse. 21 Each of four different batches of hiPSC-SIs was transplanted into two recipients. **P < 0.01 vs. 22 control. a.u., arbitrary units.

fsTo examine if islet hormone-expressing cell distribution and insulin levels can be measured in single intracameral hiPSC-SIs post-transplantation, we adopted ACE-Tx/in vitro CM and immunofluorescence labelling. Before transplantation into the ACE, a fraction of each batch of hiPSC-SIs was used as a control group. Comparison of the distribution of insulin-, glucagon- and somatostatin-immunofluorescence and quantification of the immunofluorescence intensity of insulin were carried out. Sample confocal micrographs show that a single intracameral hiPSC-SI retrieved at 90 dpt remained intact, has reasonably normal morphology and displayed satisfactory immunofluorescence labelling (Fig. 4A–C). A majority of cells in both pre-transplanted hiPSC-SIs as control and transplanted ones were insulin-positive (Fig. 4A–C). A very few cells displayed glucagon, somatostatin and insulin/glucagon immunofluorescence, respectively (Fig. 4B, C). These islet-hormone positive cells appeared randomly scattered throughout a hiPSC-SI (Fig. 4B, C). There was no marked difference in the distribution of glucagon, insulin and somatostatin immunofluorescence between control and transplanted hiPSC-SIs (Fig. 4B, C). These data together with those obtained with ACE-Tx/in vivo SM further verify that these in vitro-generated hiPSC-SIs resemble native human islets at both gross morphological and immunocytochemical levels and are suitable candidates to serve as surrogates for the native human islet. Moreover, consistent with variable light scattering signals, intense, weak or no insulin immunofluorescence appeared in different subpopulations of cells within a single intracameral hiPSC-SI (Fig. 4B, C). Importantly, immunofluorescence quantification reveals that transplanted hiPSC-SIs exhibited significant elevation in insulin immunofluorescence as compared to control hiPSC-SIs (*P* < 0.01) (Fig. 4D). These data support the notion that the observed dynamic increase of light scattering signals that occurred after transplantation is due to the upregulation of insulin secretory capacity in transplanted hiPSC-SIs. (Note: Figure 4 and its legend are missing. Please add them here!)

## Discussion

The ACE has been used as an ectopic site for transplantation followed by microimaging of native tissues in quite a few studies, but has never been used to observe in vivo survival and development of in vitro-engineered tissues including hiPSC-SIs^21,23,29–35^. Before the present work, it was questionable whether the ACE can serve as an ectopic site for transplantation and in vivo microimaging of hiPSC-SIs. This is because hiPSC-SIs generated in vitro by using currently available techniques can behave differently from native tissues in respect of survival, engraftment and growth^10–16^. The former may contain immature or undesirable cell populations and undergo heterogeneous development during post-transplantation, bringing about tumorigenic risks and other safety issues in vivo^10–16^. By carrying out the present work, we demonstrate that the ACE can competently serve as an ectopic site for transplantation and in vivo microimaging of hiPSC-SIs although they possess the above discussed peculiar properties.

We can now satisfactorily use the ACE as a transplantation and microimaging site for hiPSC-SIs, thanks to its unique optical and biological features. This ectopic site is characterized by immune privilege, which allows implanted grafts from genetically different individuals to survive for prolonged or indefinite periods of time^36–42^. It also possesses the oxygen-rich milieu and less stressful space where transplanted hiPSC-SIs are immersed in circulating aqueous humor containing relatively low concentration of glucose and effective capacity for metabolic waste disposal^21,23^. Importantly, the ACE is structured with the densely vascularized and richly innervated iris and the cornea as a front window^21,23^. Thanks to these features, we demonstrate that ACE transplanted with single hiPSC-SIs is fully qualified for serial, noninvasive in vivo microscopy at high spatiotemporal resolution over a 3-month post-transplantation period. Together as core modalities of the present method, ACE-Tx/in vivo SM, ACE-Tx/in vivo CM and ACE-Tx/in vitro CM complement each other with different capabilities. Specifically, ACE-Tx/in vivo SM is easy-to-use and cost-effective. ACE-Tx/in vivo SM and ACE-Tx/in vivo CM can effectively acquire intuitive and instantaneous results. ACE-Tx/in vivo CM and ACE-Tx/in vitro CM are characterized by high spatial resolution and reliable quantification ability, the former being featured with high temporal resolution, the latter being able to verify molecular identities at the cellular level.

Pancreatic islets naturally occur as many individual micro-organs rather than a sizable, single organ. All endocrine cells within an individual islet are morphologically and functionally integrated into a sole entity to orchestrate their performance in response to glycemic alterations^43–46^. This suggests that such a micro-organotypic structure is likely to be critical for islet differentiation and specification. The present work demonstrates that transplanted hiPSC-SIs can be arranged to desired regions of the iris where they remain largely separated and intact, and manifests that individual intracameral hiPSC-SIs can be optimally monitored by noninvasive in vivo microscopy^21^. By virtue of the same hiPSC-SI serial monitoring, ACE transplantation may lead to understanding the fine-tuned mechanisms responsible for in vivo hiPSC-SI maturation, function and survival. This in combination with the feasibility to retrieve intact single hiPSC-SIs for in vitro microscopy and other in vitro measurements may facilitate mechanistic dissection of various molecular and cellular events in transplanted hiPSC-SIs^21^.

By using the present method, we have found that hiPSC-SIs generally undergo engraftment, survival, visible growth, appreciable vascularization and marked elevation in light backscattering/insulin secretory capacity in a progressive manner when transplanted into the ACE. In fact, ACE-Tx/in vivo CM and ACE-Tx/in vitro CM confirm that overall vascularization and light backscattering significantly increase in parallel with insulin immunofluorescence over a 3-month post-transplantation. Interestingly, the in vivo dynamics of the light backscattering, which represents the abundance of mature insulin secretory granules, from intracameral hiPSC-SIs well matches the time course of the antihyperglycemic effect of hiPSC-derived insulin producing cells transplanted under the kidney capsule^6,21^. Both of them increase gradually for the first two months and then remain unaltered during the post-transplantation period^6^. This strongly indicates these two sites provide the hiPSC derivatives with a similar niche for in vivo maturation of the hiPSC derivatives. However, these in vivo events do not homogeneously occur in all transplanted hiPSC-SIs and throughout a single individual hiPSC-SI. In addition to surviving hiPSC-SIs, some develop appreciable cystic lesions or decrease in size. Moreover, an uneven distribution of light scattering signals match that of insulin immunofluorescence in hiPSC-SI cryosections. Light scattering signals are intense in most regions, but weak or not detectable in some areas or small zones. Likewise, some subpopulations of cells within a single intracameral hiPSC-SI exhibit intense immunofluorescence, whereas the others display weak or no immunofluorescence. This finding may reflect the heterogeneous tissue architecture and/or cellular make up of hiPSC-SIs and is a feature this methodology can be used to monitor for improved differentiation. Taken together with the fact that light scattering signals predominantly result from zinc-insulin crystals within insulin secretory granules in the native islet^26,27^, the findings demonstrate that intracameral hiPSC-SIs are further matured at least in their insulin secretory capacity. However, the insulin-expressing cells in the same intracameral hiPSC-SIs have different cell fates, manifested as strong, weak or no insulin immunofluorescence and perhaps cell disintegration or dedifferentiation. Mechanisms responsible for the observed heterogeneity in the engraftment, survival, growth and insulin-expressing cell fates of intracameral hiPSC-SIs requires further investigation.

The data on the presence of subpopulations of cells with low insulin secretory capacity in intracameral hiPSC-SIs may hinder clinical hiPSC-SI transplantation^10,12,14,15^. To solve it, one of the best choices may be to construct new surrogate islets by optimizing their cytoarchitecture and vasculature with highly purified, optimally differentiated hiPSC-SI cells in an ideal physiological ratio and pattern on top of the engineered 3D microvascular network. Moreover, time courses of in vivo vascularization and light scattering signals of hiPSC-SIs are intriguing and important since they are indispensable for determining time points when corresponding interventions should be applied for the best outcome.

As well known, in vivo safety and efficacy issues, like tumorigenicity, unwanted differentiation and inadequate maturation, of hiPSC derivatives including hiPSC-SIs have long stood as clinical hurdles for hiPSC therapies^10–16^. This is largely because these adverse events and their changes following intervention are hidden in hiPSC derivatives engrafted in optically inaccessible sites and can only be invasively analyzed at a single time point, but not visualized in a longitudinal, nondestructive and intravital manner with a high spatiotemporal resolution^4,6,11,17–22^. Such a long-standing dilemma calls for an urgent need for the approach described in the present work. This approach, that is, ACE approach, fits the need exactly. It allows high-resolution, noninvasive and serial in vivo tracking of the growth of hiPSC-SI grafts, whose abnormal growth echoes their tumorigenicity, and the backscattering signal of these grafts, which reflects the abundance of mature insulin secretory granules and can be used to judge the unwanted differentiation and inadequate maturation as well as cystic lesions of these grafts^21^. In addition, by monitoring changes in these two in vivo parameters subsequent to pharmacological treatment, the ACE approach can be applied as a screening tool for identifying compounds for promoting in vivo hiPSC-SI differentiation and maturation.

The present work establishes a unique high-resolution imaging method comprised of ACE-Tx/in vivo SM, ACE-Tx/in vivo CM, and ACE-Tx/in vitro CM to study cellular composition, survival, function and vascularization after transplantation. It is not only able to noninvasively and serially track the in vivo dynamics of several key parameters in an intuitive and instantaneous manner, but is also time-saving, cost-efficient and safe for in vivo evaluation of hiPSC-SIs with unreliable safety profiles^10,14–16^, thus having technical merits as a potent tool for screening for clinically appropriate hiPSC-SIs. The obtained data demonstrate that transplanted hiPSC-SIs may undergo heterogeneous cell fates, engraftment, vascularization, and insulin secretory capacity. Applying the ACE approach to genetically modified cells provides opportunity for gaining novel insights for differentiation and transplantation process optimization for better therapeutic efficacy and safety of stem cell-derived islets^3,10,12,14,47^.
